# Detection of Direction-Of-Arrival in Time Domain Using Compressive Time Delay Estimation with Single and Multiple Measurements

**DOI:** 10.3390/s20185431

**Published:** 2020-09-22

**Authors:** Youngmin Choo, Yongsung Park, Woojae Seong

**Affiliations:** 1 Department of Defense Systems Engineering, Sejong University, Seoul 05006, Korea; ychoo@sejong.ac.kr; 2 Scripps Institution of Oceanography, University of California San Diego, La Jolla, CA 92093-0238, USA; yongsungpark@ucsd.edu; 3 Department of Naval Architecture and Ocean Engineering, Research Institute of Marine System Engineering, Seoul National University, Seoul 08826, Korea

**Keywords:** compressive sensing, time delay estimation, direction-of-arrival, SAVEX15

## Abstract

The compressive time delay estimation (TDE) is combined with delay-and-sum beamforming to obtain direction-of-arrival (DOA) estimates in the time domain. Generally, the matched filter that detects the arrivals at the hydrophone is used with beamforming. However, when the ocean noise smears the arrivals, ambiguities appear in the beamforming results, degrading the DOA estimation. In this work, compressive sensing (CS) is applied to accurately evaluate the arrivals by suppressing the noise, which enables the correct detection of arrivals. For this purpose, CS is used in two steps. First, the candidate time delays for the actual arrivals are calculated in the continuous time domain using a grid-free CS. Then, the dominant arrivals constituting the received signal are selected by a conventional CS using the time delays in the discrete time domain. Basically, the compressive TDE is used with a single measurement. To further reduce the noise, common arrivals over multiple measurements, which are obtained using the extended compressive TDE, are exploited. The delay-and-sum beamforming technique using refined arrival estimates provides more pronounced DOAs. The proposed scheme is applied to shallow-water acoustic variability experiment 15 (SAVEX15) measurement data to demonstrate its validity.

## 1. Introduction

To detect a target signal, the sonar system (equivalent to the radar system in the air) uses an array that consists of multiple hydrophones, enabling target localization via direction-of-arrival (DOA) detection. The DOAs are estimated using the arrival phase difference occurring across the hydrophones within the array. An appropriate phase shift introduced to each hydrophone output signal creates in-phase waveforms, thereby strengthening the sum of the received signals, and indicating the DOAs corresponding to the introduced phase shift [1]. The previously mentioned conventional beamforming (i.e., delay-and-sum beamforming in the frequency domain) suffers from low-resolution DOA results owing to the limited number of sensors in the array. To overcome this problem, adaptive beamforming schemes, such as minimum variance distortionless response (MVDR) [1,2] and multiple signal classification (MUSIC) [1,3], are applied. However, their applications are restricted by insufficient measurement data as well as coherent arrivals that typically exist underwater.

Recently, compressive sensing (CS) has been combined with beamforming [4,5,6,7,8] to solve an underdetermined linear system y=Ax [9,10]. In the conventional compressive beamforming technique [6,7], y is the array-captured measurement vector in the frequency domain and is identical to the data used in classical beamforming schemes; x is an unknown vector to be solved by CS, whose entries correspond to the amplitudes of the arrival angles in predefined grids; and A is the sensing matrix that shows the linear relation between x and y, and is composed of replicas (i.e., simulated pressures at the sensors with plane-wave approximation) for the predefined arrival angles as columns of A. In general, the dimension of y is less than that of x, leading to an underdetermined linear system with infinite solutions. However, by exploiting the sparsity of the target signals underwater, the linear system can be solved with CS using the sparsity-promoting objective function.

The compressive beamforming technique provides high-resolution DOA estimates with limited observation [6,7]. Its robustness-to-noise performance can be improved by treating multiple measurements over time [5,8]. When the actual DOA does not match one of the predefined arrival angles (basis mismatch), it is detrimental to compressive beamforming performance [7,11]. To mitigate basis mismatch, grid-free CS adopting atomic norm with the dual problem [12,13,14,15] is applied for beamforming [16,17]. Then, the higher resolution DOAs are found in the continuous angle domain.

The previously mentioned beamforming schemes are conducted in the narrowband frequency domain and their performance degrades with the large aperture array, owing to the spatial aliasing. In this work, time domain beamforming is used to exploit broadband-transmitted sonar waveforms such as the linear frequency modulated (LFM) pulse [1]. During time domain beamforming, the matched filter (MF) is applied to compress the pulse of the LFM signal, thereby providing an approximate channel impulse response (CIR). The CIRs at the array are combined with the delay-and-sum beamforming in the time domain, adding different delays (in the present work, delays are interchangeable with time delays) to the received signals at the array. The delay amount is related to the DOA. The result from time domain beamforming indicates the DOAs according to the arrival travel times. However, when ocean noise contaminates the data, blurring is observed around the DOAs in the beamforming result, consequently preventing unambiguous DOA detection.

For clear DOA detection, refined CIRs are required and CS is applied for the refinement by its sparse channel estimation [18,19,20]. In underwater acoustics, a CS-based CIR estimation was introduced in reference [21], where channel parameters, including amplitudes and time delays of arrivals in CIRs, were evaluated using orthogonal matching pursuit (one of the representative CS solvers) after modification. As in the narrowband DOA estimation using CS, the CS-based CIR estimation suffers from basis mismatch. Grid-free CS has recently been used to estimate the CIR in the continuous time domain, showing better performance in terms of noise reduction and resolution [22]. For the present work, grid-free compressive time delay estimation (TDE) was extended for its application to the array data, and reliable performance was demonstrated. Unlike reference [22], two different CS schemes (off- and on-grid CS schemes) were sequentially used to extract the CIRs from the measurement data at the array to avoid the use of the manually determined noise bound related to the SNR for array data, since the hand-operated noise bound made the practical application of grid-free TDE estimation difficult. First, arrival time delays were estimated in the continuous time domain; then, the dominant arrivals constituting the received signal were chosen in the discrete time domain by exploiting the grid-free CS result from the previous step. Owing to this selection, minor noise should be suppressed, resulting in more obvious DOAs. To increase the robustness of the grid-free compressive TDE, the multiple measurement method was used; the grid-free compressive TDE was modified to search the common arrival time delays over multiple measurements. Then, the CIRs along the sensors in the array were used with the delay-and-sum beamforming in the time domain to detect DOAs clearly.

Section 2 introduces theoretical background of the current work, including MF and conventional (on-grid) and advanced (off-grid) CS application to CIR estimation, and shows the beamforming results with CS which are compared with those from the MF, using the synthetic data from the simulation. The grid-free compressive TDE for single measurement is modified to exploit multiple measurements in Section 3. A simple numerical experiment is conducted to examine the performance of extended grid-free compressive TDE in terms of suppressing noise. The common time delays over measurements from the extended grid-free compressive TDE result in more refined CIRs, which are beneficial to DOA detection. Section 4 applies the time domain beamforming schemes to the low- and high-frequency measurement data obtained from the shallow-water acoustic variability experiment 15 (SAVEX15). Finally, in Section 5, the present work is summarized.

## 2. DOA Detection in the Time Domain Using Single Measurement

Acoustic waves emitted from an underwater transmitter travel through a water medium bounded by the sea surface and the sea bottom. Acoustic waves traveling via various propagation paths arrive at the receiver, such as a hydrophone, with different time delays and directions. The received signal can be thought of as a linear combination of the source waveforms with different time delays, and it is denoted as follows:

(1) $$r\left(t\right)=\sum_{k=1}^{K}a_{k}s\left(t\;-\;\tau_{k}\right),$$

where $a_{k}$ is the amplitude corresponding to the time delay $\tau_{k}$, and K is the total arrival number. In this work, it is assumed that the waveform is distorted insignificantly during the propagation, owing to its weak dependence on the frequencies belonging to the source spectrum $\hat{s}\left(\omega \right)$. The ideal CIR corresponding to the acoustic signal is expressed as $h\left(t\right)=\sum_{k=1}^{K}a_{k}\delta \left(t\;-\;\tau_{k}\right)$, where δ(t) is the Kronecker delta.

The CIR clearly shows the arrivals and the CIRs along the depth indicate arrival structures that are related to DOAs. The DOAs can be estimated using the beamforming schemes with acoustic signals (or the CIRs) measured by the multiple hydrophones in an array. In this work, a conventional delay-and-sum beamformer is used with the CIRs to detect the DOAs in the time domain. While the MF is generally used to evaluate the (approximate) CIRs, CS is applied to obtain clearer DOAs.

### 2.1. The MF and Its Application to DOA Estimation

To estimate the CIR, the $a_{k}$ and $\tau_{k}$ in Equation (1) should be extracted from the measurement data. This is difficult in practice, owing to the masking of the signal by the noise. Alternatively, the MF is used to find arrivals when a transmitted signal from the source is known. This is achieved using the cross-correlation of the known source signal with the received signal, as follows:

(2) $$\tilde{r}\left(t\right)=\sum_{k=1}^{K}a_{k}\tilde{s}\left(t\;-\;\tau_{k}\right),$$

where $\tilde{s}\left(t\right)$ is the source–waveform autocorrelation. The signal-to-noise ratio (SNR) is improved by the MF. In particular, when the LFM pulse waveform is used as the transmitted signal, the MF provides sharp peaks corresponding to the arrivals as the ideal CIR (i.e., pulse compression).

**Figure 1:**
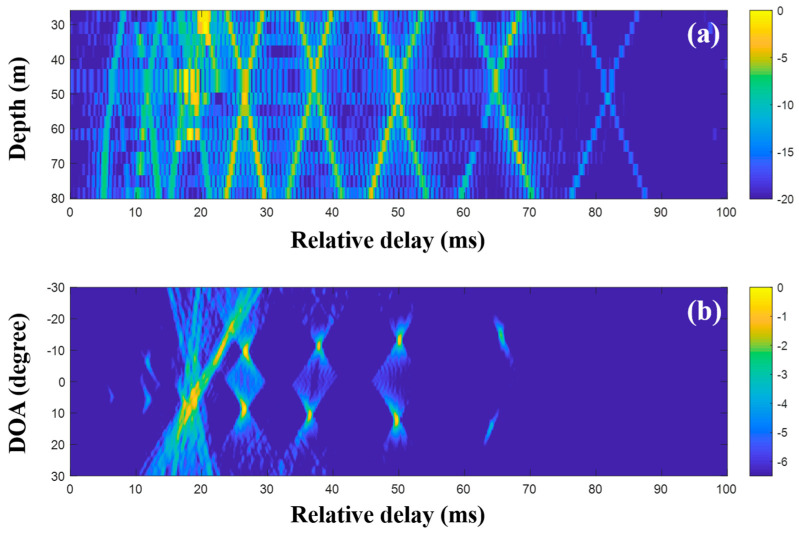
Direction-of-arrival (DOA) estimation using the matched filter (MF) with a single measurement. Noise is absent: (**a**) arrival structures via application of MF to the signals at the vertical line array (VLA) and (**b**) DOAs using delay-and-sum beamforming with the arrival structures.

Figure 1 shows the MF results according to the vertical line array (VLA) sensors and the DOAs in the time domain using the MF results. The simulation is conducted with the SAVEX15 environment [23,24], where an LFM signal from 0.5 to 2.0 kHz is transmitted from the source. The geoacoustic parameters, including density, sound speed, and attenuation of the sediment, are chosen in reference to the clay and silt, which cause high-reflection loss. Therefore, the acoustic signals from the simulation can be considered as sparse signals. Reference [23] describes the simulation geometry in detail. In this work, a conventional ray model [1] was used for the numerical experiment for the convenience of analysis, owing to its clear indication of arrival histories. Furthermore, with the ray model, it is easy to implement a Doppler shift in arrival signals according to their paths. Its effect on the estimation of CIRs will be examined in a later simulation (refer Section 3.2).

Distinctive arrivals are obtained from the MF using the noise-free received signals, and these clearly form the x-shape arrival structures in the later CIRs. The conventional beamforming clearly detected the DOAs with the CIRs; in this work, all the CIRs and beamforming results were normalized with their maximum values, and upward and downward incoming acoustic signals to the VLA had negative and positive signs, respectively. Notably, the high-intensity earlier arrivals were observed owing to the refracted (or trapped) rays within the sound channel that resulted from the SAVEX15 sound speed profile. These are of less interest in this work.

**Figure 2:**
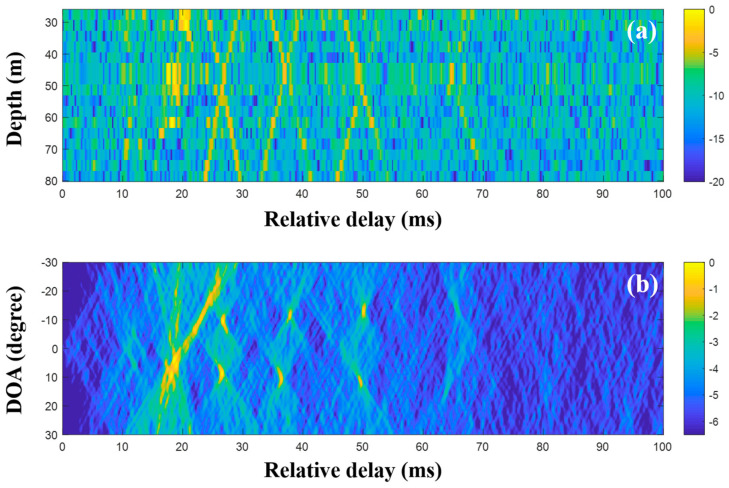
Direction-of-arrival (DOA) estimation using the matched filter (MF) with a single measurement. The signal-to-noise ratio (SNR) is −9.8 dB: (**a**) arrival structures via application of the MF to the signals at the vertical line array (VLA) and (**b**) DOAs using delay-and-sum beamforming with the arrival structures.

The actual measurement data include noise, which masks the arrivals; a white Gaussian noise (*n(t)*) was added to Equation (1) for later simulation. While the MF is useful for the noise reduction, it was not sufficient to remove the noise clearly. The MF was applied to the noisy data, as shown in Figure 2a. The SNR of the simulation is −9.8 dB (in the decibel scale), which is defined as SNR = $\sum_{j=1}^{J}\int_{\omega_{L}}^{\omega_{H}}{\left|{\hat{r}}_{j}\left(\omega \right)\right|}^{2}d\omega /\sum_{j=1}^{J}\int_{\omega_{L}}^{\omega_{H}}{\left|{\hat{n}}_{j}\left(\omega \right)\right|}^{2}d\omega$. The ${\hat{r}}_{j}\left(\omega \right)$ and ${\hat{n}}_{j}\left(\omega \right)$ are spectra of the received signal and noise at the *j*th hydrophone at the array composed of *J* hydrophones, respectively; $\omega_{L}$ and $\omega_{H}$ are the lowest and highest angular frequencies of the source spectrum, respectively, corresponding to 0.5 and 2 kHz in this simulation. After the MF application, the arrivals were revealed to be less clear compared with those of Figure 1a. In particular, the earlier and later arrivals were smeared by the random Gaussian noise. 

During the beamforming, random directional noise was diminished and the actual arrivals corresponding to the specific directions were strengthened, as shown in Figure 2b. In contrast to Figure 1b, however, the blurred patterns emerged around the DOAs, which should be even more prevalent in the real data, increasing the ambiguities in the DOA detection. The purpose of this work is noise suppression in DOA estimation using CS, which will be subsequently explained.

### 2.2. Compressive TDE and Its Application to DOA Estimation

Regarding the MF, instead of the detection of the $a_{k}$ and $\tau_{k}$ of the arrivals, the uncorrelated noise with the source waveform was suppressed by cross-correlation processing. Alternatively, the compressive TDE extracted the $a_{k}$ and $\tau_{k}$ from the measurement data using an optimization method, where an objective function that promotes the arrival sparsity was used. To apply conventional CS for this extraction, Equation (2) was approximated as follows [25]:

(3) $$\tilde{r}\left(t\right)\approx \sum_{n=1}^{N}x_{n}\tilde{s}\left(t\;-\;n∆\tau \right).$$

The time delays of the candidates for the actual arrivals were evenly discretized with the grid of ∆τ, and the linear combination of the candidates (i.e., source waveform autocorrelations with the time delays n∆τ) was used to represent the received signal. In general, for this representation, *N* is much larger than *K*. When n∆τ is equal to $\tau_{k}$, the $x_{n}\;$ comprises the non-zero value equal to $a_{k}$; otherwise, $x_{n}$ is zero. Equation (3) was rearranged using the vector and matrix notations for CS, as follows:

(4) $$\tilde{r}\;\approx Ax.$$

A component of the measurement vector $\tilde{r}$ is $\tilde{r}\left(t_{j}\right)$, corresponding to the received signal at time $t_{j}$. A column of the sensing matrix A is the delayed source waveform autocorrelation with n∆τ along the time series, and its amplitude is $x_{n}$, which consists of the unknown vector x. When the actual arrival time delays fall on the grids, only *K* elements out of the *N* elements in x have non-zero values, which can be considered as sparse. Therefore, CS can be applied to the linear system as Equation (4) to reconstruct the sparse signal, as follows:

(5) $$\underset{x\in {\mathbb{R}}^{N}}{\min}{∥x∥}_{1}\;\mathrm{subject}\;\mathrm{to}\;∥\tilde{r}\;-\;A{x∥}_{2}<\varepsilon ,$$

where ε reflects the noise effects on the received signal and is practically unknown. Originally, the minimization of the *l_0_*-norm of x, for which the number of non-zero elements in x is counted, is used; however, this is computationally infeasible [9,10]. Instead, the relaxed minimization of *l_1_*-norm of the x with the assumption of a sufficient sparse signal was used, and this can be calculated with a convex optimization algorithm such as the MATLAB cvx tool [26]. 

Equation (5) is the standard approach for CS-based recovery of the CIR from the measurement data. To properly approximate Equation (2) using Equation (3), however, a fine grid with a small ∆τ is required; this results in the increase in the number of columns in A as well as column similarity (i.e., coherence), and these are detrimental to the sparse recovery condition [7,11]. When ∆τ increases, the arrival time delays fall off the coarse grids; this is called a basis mismatch, which deteriorates CS performance.

In this work, to overcome basis mismatch, CS was sequentially used. In fact, the arrival time delays are in the continuous time domain. First, arrival candidate time delays were searched for in the continuous time domain using grid-free CS [22]. In this step, the unknown signal noise was ignored. The number of candidates from grid-free CS is much smaller than those from Equation (5) with the small grid. Then, conventional CS was used with the reduced form of the candidate set in the discrete time domain to recover the contributing arrivals for the received signal.

#### Grid-Free Compressive TDE

To find the arrivals in the continuous time domain, the total variation (TV) norm (or atomic norm) was introduced. This corresponds to the *l_1_*-norm in the discrete time domain and measures the sparsity of the continuous signal [13,16,17]. The sparsity was imposed on the CIR h(t) with the TV norm, while it is expressed as $\hat{h}\left(\omega \right)=\;\sum_{k=1}^{K}a_{k}e^{-j\omega \tau_{k}}$ in the frequency domain. These were used as the objective function and the regularization in grid-free CS, respectively, as follows [16]:

(6) $$\underset{x}{\min}{∥x∥}_{TV}\;\mathrm{subject}\;\mathrm{to}\;\{\begin{array}{c} \hat{h}=F_{M}x+{\hat{n}}_{n}\\ ∥{{\hat{n}}_{n}∥}_{2}\leq \delta \end{array},$$

where x is the set of arrival amplitudes, ${∥x∥}_{TV}=\sum_{k=1}^{K}\left|a_{k}\right|$, and $F_{M}$ is the Fourier-transform matrix consisting of $e^{-j\omega_{m}\tau_{k}}$, where $\omega_{m}$ is an angular frequency belonging to the source spectrum. A component of the measurement vector $\hat{h}\in {\mathbb{C}}^{M}$ corresponds to the Fourier-transform of the h(t) at $\omega_{m}$ (i.e., $\hat{h}\left(\omega_{m}\right)$) that was calculated with the $\hat{r}\left(\omega_{m}\right)/\hat{s}\left(\omega_{m}\right)$ for the present work. The dimension *M* of $\hat{h}$ is the number of the source spectrum angular frequencies used for arrival recovery. An element of ${\hat{n}}_{n}\in {\mathbb{C}}^{M}$ is the normalized noise spectrum that is represented as $\hat{n}\left(\omega_{m}\right)/\hat{s}\left(\omega_{m}\right)$. The δ is the noise bound, which is unknown as is the case for ε in Equation (5). Note that the dimension of x to be recovered by CS is infinite, because it is defined in the continuous time domain. To solve the primal optimization problem with the infinite dimension, its dual optimization problem was exploited.

In the dual problem, the dual function g(c,ξ) is defined as the minimum value for the Lagrangian of Equation (6), as follows [22,27]:

(7) g(c,ξ)=infx{∥x∥TV+Re[cH(h^−FMx−n^n)]+ξ(n^nHn^n−δ2)},

where c and ξ are the Lagrange multipliers for the equality and inequality constraints of Equation (6), respectively, and the superscript *^H^* denotes the Hermitian. The last term accounts for the noise in the signal with the noise bound δ. A proper choice of the δ controlling signal sparsity is crucial for recovering the arrivals exactly from the signal [22]; however, it is difficult to determine the δ in practice. In this work, instead of the perfect recovery of the arrivals with the proper δ, the major arrivals constituting the signal were estimated with sequential CS usage. In the first step, noise is neglected (i.e., δ=0), leading to the number of arrivals being greater than that of the true arrivals due to overfitting to the signal. Equation (7) was rearranged without the noise term, as follows:

(8) g(c)=Re[cHh^]+infx{∑k=1K[|ak|−Re[(FMHc)kHak]]},

where ${\left(F_{M}^{H}c\right)}_{k}$ is the *k*th component of the $F_{M}^{H}c$ (i.e., the inner product of the *k*th column of the $F_{M}$ and c). Consider the term multiplied by $\left|a_{k}\right|$ in the infimum; it is always larger than $\left[1-\left|{\left(F_{M}^{H}c\right)}_{k}\right|\right]$. When $\left[1-\left|{\left(F_{M}^{H}c\right)}_{k}\right|\right]$ is greater than zero, its minimum value becomes zero; otherwise, it goes to the undesirable −∞. The dual problem was derived by maximizing the dual function over the c, leading to the equation using the previously mentioned property, as follows:

(9) maxc∈ℂMRe[cHh^] subject to ∥FMHc∥∞≤1.

Equation (9) can be recast as semidefinite programming, as follows [13,15]:

(10) maxc∈ℂMRe[cHh^] subject to [QccH1]≽0, ∑i=1M−jQi,i+j={1,0, j=0,j=1,2,⋯,M−1,

where $Q_{i,j}$ is an element of the M×M matrix Q. Thus, the dual problem can be solved using semidefinite programming, which is one of the convex optimization problems [26]. Here, the MATLAB cvx tool was used to obtain the solution for Equation (10). Note that c was obtained from the dual problem; it is directly associated with the time delay (τ) of the arrival that satisfies the following equation: $\left|F_{M}^{H}c\right|=\left|\sum_{m=0}^{M-1}c\left(\omega_{m}\right)e^{-j\omega_{m}\tau }\right|=1$.

The arrival time delays were calculated using grid-free CS (i.e., grid-free compressive TDE) that becomes the set of candidate time delays for the actual arrivals at the second step. These were used to estimate the corresponding amplitudes using the pseudoinverse matrix with $\hat{h}=F_{M}x$ [16]. However, the recovered arrivals contained spurious arrivals, owing to the noise. Here, to suppress the noise, conventional CS was used with a slight modification of Equation (3). The time delays were used for the CS matrix in the second step. Again, the signal after the cross-correlation with the transmitted signal was approximated as follows:

(11) $$\tilde{r}\left(t\right)\approx \sum_{n\in n_{c}}x_{n}\tilde{s}\left(t-n∆\tau_{c}\right).$$

At this time, $∆\tau_{c}$ is a sampling time step for the signal (i.e., 1/sampling frequency), and a component of $n_{c}$ is the positive integer nearest to $\tau /∆\tau_{c}$. $x_{n}$ is the corresponding amplitude to be recovered. The delayed source waveforms of Equation (11) are the columns of the CS matrix for the second step. CS was applied with a much smaller candidate set compared with that of Equation (3) to estimate the $x_{n}$. Here, the multipath matching pursuit (MMP), which is the expansion of the orthogonal matching pursuit (OMP) [28], was used to effectively solve the conventional CS in the second step with the predefined parameter $K_{c}$, which is the desired number of arrivals extracted from the data. $K_{c}$ arrivals out of all the arrivals from grid-free CS comprise the non-zero amplitudes, which should be the contributing arrivals to the signal. By selecting the major arrivals from the data, the arrivals appear with less noise, thereby improving DOA detection.

The steps for the arrival extraction are summarized as follows. First, grid-free CS was applied to the measured data (obtained from the experiment). The δ reflecting the noise effect was set to zero. The arrival time delays, including the actual and spurious arrivals, were evaluated from the grid-free compressive TDE. Next, the sensing matrix, consisting of the delayed source waveform autocorrelations with the time delays as its columns, was used for conventional CS, with the desired number of arrivals, $K_{c}$. From sequential CS usage, $K_{c}$ arrivals can be obtained, and these should be the major arrivals that contribute to the signals.

**Figure 3:**
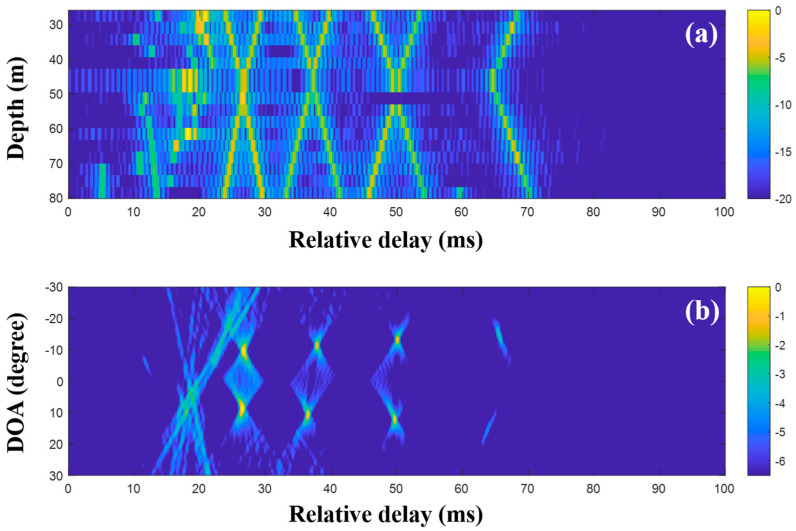
Direction-of-arrival (DOA) estimation using compressive sensing (CS) with a single measurement. Noise is absent: (**a**) arrival structures via sequential CS usage with the signals at the vertical line array (VLA) and (**b**) DOAs using delay-and-sum beamforming with the arrival structures.

Figure 3 shows the arrival structures using sequential CS with the synthetic data at the VLA (identical to that of Figure 1), as well as the corresponding DOAs from the delay-and-sum beamforming with the arrival structures. For the simulation of this work, the desired arrival number, $K_{c}$, was set to 10, and is the same for the following simulations. Note that some arrivals (in particular, the earlier messy and later weak arrivals) were not detected during the selection of the contributing arrivals, and the associated DOAs are not evident or are less apparent. Nevertheless, CS clearly captured the arrivals, forming the x-shape. When the data are measured with high SNR values, MF usage is preferable, owing to the clear arrival appearance and lower computational burden.

**Figure 4:**
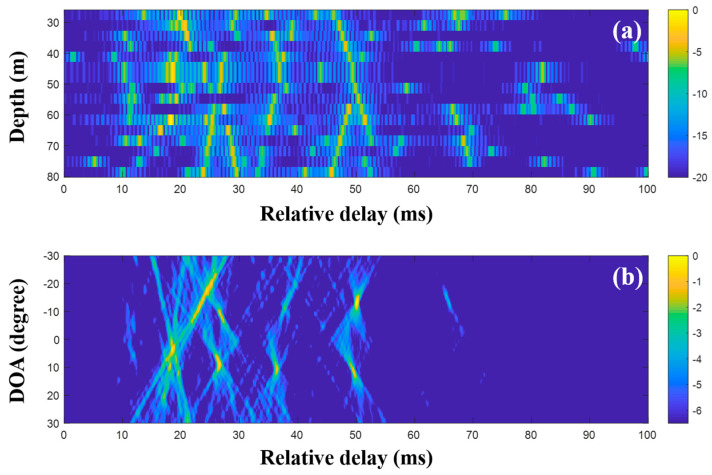
Direction-of-arrival (DOA) estimation using compressive sensing (CS) with a single measurement. The signal-to-noise ratio (SNR) is −9.8 dB: (**a**) arrival structures via sequential CS usage with the signals at the vertical line array (VLA) and (**b**) DOAs using delay-and-sum beamforming with the arrival structures.

Consider noise-contaminated data. Figure 4 shows the arrival structures obtained from CS and the corresponding DOAs, where the same data for Figure 2 were used. While the fictitious arrivals appeared randomly and many of the actual arrivals were missed owing to the noise, the undesired signals produced near the actual arrivals were less than the MF result. During the beamforming process, the random arrivals were suppressed, whereas the DOAs for the actual arrivals were enhanced. The DOAs were detected with less ambiguity but at the cost of losing the weak arrivals and the corresponding DOAs. 

This problem can be overcome by the exploitation of multiple measurements over time, with the assumption of steady arrivals during the measurement; this is discussed in the following sections.

## 3. DOA Detection in the Time Domain Using Multiple Measurements

### 3.1. Conventional Exploitation of Multiple Measurement for DOA Estimation

During the operation of the sonar system for target searching or CIR measurements over time for underwater communication, the source waveform is transmitted repeatedly with a specific period of time. The VLA-measured CIRs can be considered time-invariant, owing to the calm sea environment or a brief observation time. The steady arrivals during the measurement can be exploited to suppress the noise for both the MF and CS.

For the MF, the cross-correlation between the source and received signals was applied to each measurement (i.e., to each snapshot). Then, the MF results over the multiple measurements were averaged. The regular arrivals tend to be enhanced by the averaging process, whereas random noise should be reduced.

**Figure 5:**
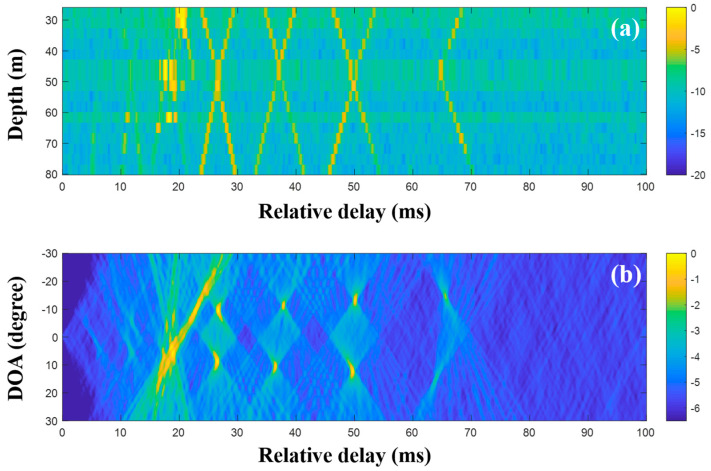
Direction-of-arrival (DOA) estimation using the matched filter (MF) with multiple measurements. The signal-to-noise ratio (SNR) is −9.8 dB: (**a**) arrival structures via the application of the MF to the signals at the vertical line array (VLA) and (**b**) DOAs using delay-and-sum beamforming with the arrival structures.

Figure 5 shows the arrival structures acquired by the MF with multiple measurements and the corresponding DOAs. In the simulation, Gaussian noise was generated randomly for each snapshot. As expected, the arrival structures are more apparent, and the performance of the DOA estimation is enhanced. However, the weak blurred patterns still remain around the arrivals and DOAs, which should be more apparent in the real data.

**Figure 6:**
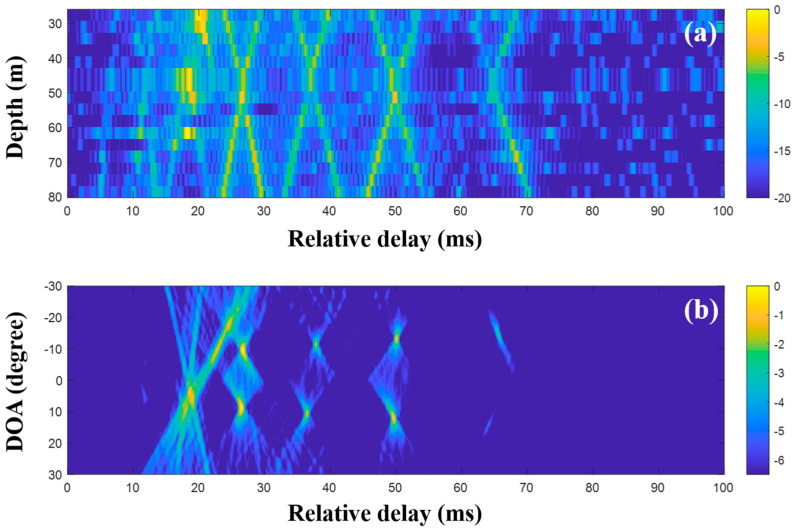
Direction-of-arrival (DOA) estimation using compressive sensing (CS) with multiple measurements. The signal-to-noise ratio (SNR) is −9.8 dB: (**a**) arrival structures via sequential CS usage with the signals at the vertical line array (VLA), and (**b**) DOAs using delay-and-sum beamforming with the arrival structures.

Like the MF, CS using a single snapshot was applied to each measurement to extract the main arrivals in the data, which were averaged over the multiple snapshots. Figure 6 shows the arrival structures via CS with the multiple measurements and corresponding DOAs. Note that the missed parts of the actual arrivals in Figure 4a were recovered, and the arrival structures appear clearly. Several scattered arrivals due to the noise were diminished by the averaging, making the actual arrivals more salient. 

When the multiple measurement data are available, it is beneficial to both the MF and CS for noise suppression. Simple averaging improves DOA detection by reducing the random noise in the process of the arrival estimation. The multiple measurement technique seems to be more effective for CS because the irregular noise signals were further weakened by averaging in the CS.

### 3.2. Multiple Measurement for Grid-Free Compressive TDE: Common Time Delay

Another method can be applied with CS for the use of the multiple measurement data. When the CIRs are consecutively measured for a brief duration, the arrivals from the source are almost identical over the measurement time, and their time delays during the measurement are nearly constant. To exploit the commonality of the time delays in the multiple measurement data, the grid-free TDE for the single snapshot is expanded to handle the multiple measurement data, as follows:

(12) $$\underset{X}{\min}{∥X∥}_{gTV}\;\mathrm{subject}\;\mathrm{to}\;\{\begin{array}{c} {\hat{h}}_{1}=F_{M}x_{1}\\ {\hat{h}}_{2}=F_{M}x_{2}\\ \vdots \\ {\hat{h}}_{L}=F_{M}x_{L} \end{array},$$

where X is the set of arrival amplitudes over the multiple measurements, which is composed of the amplitudes for each single snapshot ($x_{l}$) as its column; and ${\hat{h}}_{l}$ is the transfer function for the *l*th snapshot. Further, ${∥X∥}_{gTV}$ imposes arrival sparsity over the measurement and is defined as follows [15]: ${∥X∥}_{gTV}=\sum_{k=1}^{K}{∥x_{k:}∥}_{2}$, where $x_{k:}$ is the *k*th row of X corresponding to the amplitude of the stationary *k*th arrival in the multiple snapshots. As was previously applied, noise is not considered in the first step, and the dual function for Equation (12) is denoted as follows:

(13) g(c1,c2,⋯,cL)=infX{∥X∥gTV+Re[∑l=1LclH(h^l−FMxl)]},

where $c_{1,2,\cdots ,L}$ is the Lagrangian multiplier at each snapshot. Equation (13) was rewritten to obtain its infimum, as follows:

(14) g(c1,c2,⋯,cL)=Re[∑l=1LclHh^l]+infX[∑k=1K{∑l=1L|xkl|2−Re[∑l=1L(FMHcl)kHxkl]}],

where $x_{kl}$ is the *k*th arrival for the *l*th snapshot (i.e., the *l*th component of $x_{k:}$). Consider the term inside the summation over the *k*, which can be stated as xk:xk:H−Re[αkHxk:T] for the fixed *k*. The superscript *^T^* denotes the transpose. The *l*th component of the $\alpha_{k}$ corresponds to the ${\left(F_{M}^{H}c_{l}\right)}_{k}$. When $\sum_{l=l}^{L}{\left|{\left(F_{M}^{H}c_{l}\right)}_{k}\right|}^{2}\leq 1$ (or $\alpha_{k}^{H}\alpha_{k}\leq 1$), the term in the brace (or the term inside the summation over the *k*) is always larger than zero, and its infimum becomes zero; otherwise, it becomes −∞. Thus, the dual problem is denoted as follows:

(15) maxc1,2,⋯,L∈ℂMRe[∑l=1LclHh^l] subject to supk∑l=lL|(FMHcl)k|2≤1.

Equation (15) can be restated to find its solution using semidefinite programming, as follows [14]:

(16) maxc1,2,⋯,L∈ℂMRe[∑l=1LclHh^l] subject to [QCCHI]≽0, ∑i=1M−jQi,i+j={1,0, j=0,j=1,2,⋯,M−1,

where C is the M×L matrix, which consists of $c_{l}$ as its column; I is the L×L identity matrix; and the convex optimizer cvx [26] provides the Lagrangian multipliers $c_{1,2,\cdots ,L}$. The multipliers are used to find the common arrival time delays, which satisfy the equation as follows: $\sum_{l=l}^{L}{\left|{\left(F_{M}^{H}c_{l}\right)}_{k}\right|}^{2}=1$. Recall that $F_{M}$ is a function of time delay.

**Figure 7:**
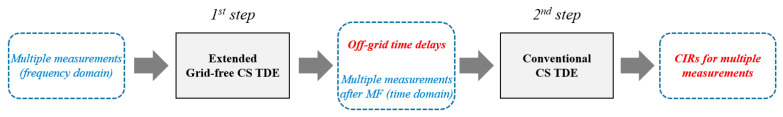
The process of extracting CIR from multiple measurements using the extended grid-free compressive time delay estimation (TDE) with conventional on-grid compressive TDE: Multiple measurements (frequency responses of received signals at a sensor) are used as input for extended grid-free compressive time delay estimation, which estimates time delays of arrivals in continuous time domain. The off-grid time delays from the previous step and matched filter (MF) results are used as input for the second step (conventional on-grid TDE). It evaluates approximated CIRs composed of dominant arrivals along multiple measurements, and their average provides refined CIR at the sensor.

The time delays from the grid-free compressive TDE with multiple measurements are those of the candidates for the contributing arrivals at the second step, and they were used with Equation (11) to obtain the major arrivals for each snapshot; then, the arrivals were averaged. During this process, the actual arrivals that were in common were enhanced, whereas the random noise arrivals were suppressed. In the previous method using multiple measurement CS, the time delays were estimated using the grid-free compressive TDE for each snapshot; here, the grid-free compressive TDE was expanded to find the common time delays over the multiple measurements. The process of extracting CIR from multiple measurements using the extended grid-free compressive TDE with conventional on-grid compressive TDE is summarized in Figure 7.

**Figure 8:**
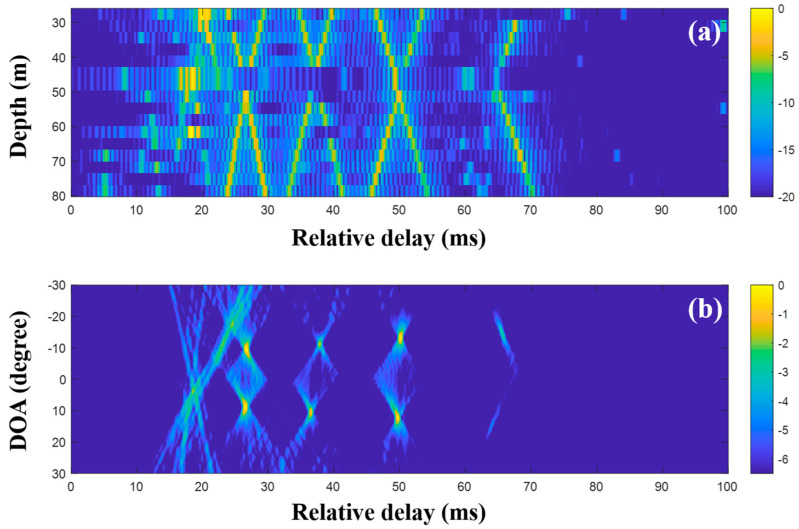
Direction-of-arrival (DOA) estimation using expanded compressive sensing (CS) with multiple measurements. The signal-to-noise ratio (SNR) is −9.8 dB: (**a**) arrival structures via sequential CS usage with the signals at the vertical line array (VLA). The compressive time delay estimation (TDE) is expanded to find the common time delays of the arrivals over the multiple measurements. (**b**) DOAs using delay-and-sum beamforming with the arrival structures.

Figure 8 shows the arrival structures using the expanded grid-free compressive TDE. While parts of the arrivals were missed during the process, the noise was removed more effectively, and the arrival structures are more clearly evident compared with those of Figure 6. It is helpful to exploit the commonality in the multiple measurements to suppress the unwanted random signals, thereby improving the DOA detection remarkably.

### 3.3. Examination of CIR Estimation Using Extended Grid-Free Compressive TDE

**Figure 9:**
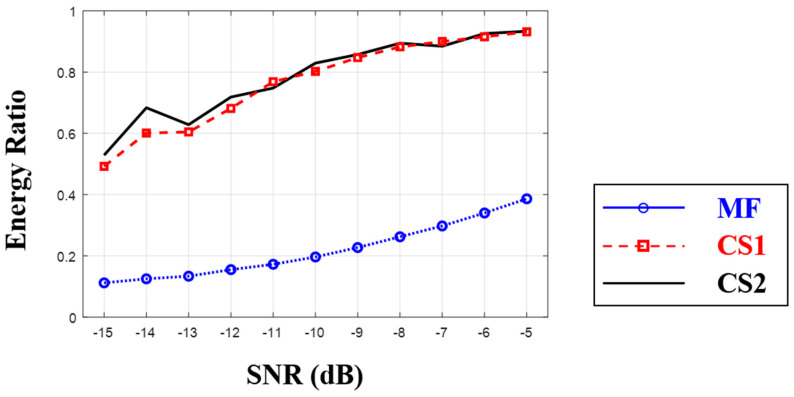
Energy ratios from three different schemes: Matched filter (MF, dotted line), repeated usage of compressive sensing (CS) along multiple measurements (CS1, dash line), and expended CS for multiple measurements (CS2, solid line). While the MF result increases with the increment of signal-to-noise ratio (SNR), it is much less than 1, owing to overall residing peaks by noise. Two CS-based schemes show similar performance for the simple environment having a single arrival. However, expended CS for multiple measurements is better at most SNRs (in particular, at low SNRs). Both CS results approach to one since their estimates are more concentrated around true arrival with the increment of SNR.

To examine the suggested CIR estimation, a simple numerical experiment is conducted, where the low frequency LFM signal (0.5–2 kHz) is transmitted through the single path and arrives at a receiver with a time delay of 0.05 s. The received signal length is 0.2 s, which is discretized with the sampling frequency of 10 kHz; the total number of signal samples is 2001. Noise is added to the noise-free signal and the aforementioned three schemes are applied to estimate a CIR with five measurements. Whereas the exact CIR is composed of a single peak at 0.05 s corresponding to the true arrival time, the estimated CIR has other peaks owing to the noise. Here, the performance of the scheme is measured with ratio of CIR energy around the true arrival to total CIR energy according to SNR; here, the CIR from 0.045 to 0.055 s is used for the energy around the true arrival, and the ratio approaches to one when the dominant peaks from the scheme are near the true time delay. The experiment is repeated 30 times at a specific SNR and the mean experimental results are shown in Figure 9.

Ratios from all schemes increase with the increment of SNR. MF suffers from overall residing noise (in particular, at low SNR), which is suppressed by the advanced schemes using CS. While two CS-based CIR estimators (the desired number of arrivals is set to five) show similar performance in the simple experiment with the single arrival, the CIR estimator exploiting the common time delays over the multiple measurements are slightly better at most SNRs (in particular, at low SNRs). Both ratios approach one as SNR increases.

Noise suppression by CS is achieved at the cost of computational burden. MF estimates the CIR with a computational time of 0.005 s in a computer with an Intel(R) Core (TM) i7-7800X CPU. The convex optimization in CS requires much larger computational time. In particular, the CIR estimator using the grid-free compressive TDE for each snapshot finds the sparse solutions along the multiple measurements, which results in a huge computational time of 670 s. The other estimator using CS searches the common spare solution over the multiple measurements and has a lower computational time of 101 s (similar to the computational time of grid-free compressive TDE using a single measurement), but still much larger than that of MF. The computational time could be reduced by using a solver such as the alternating direction method of multipliers (ADMM) [29], which trades accuracy with computational burden; this is beyond the current work’s scope.

### 3.4. Frequency Shift of a High Frequency Source

When a high-frequency source signal is transmitted during the measurement, a frequency shift is introduced in arrival signals owing to source/receiver and surface movements, and the shift depends on the receiver depth and arrival path (i.e., Doppler effect). Unlike the previous simulations, frequency-shifted source waveforms are used in Equation (1): An 11–31 kHz LFM signal, which is the same as the high-frequency source in SAVEX15, is used as the source for high-frequency synthetic data and it is randomly shifted between −500 and +500 Hz in frequency according to each snapshot in the multiple measurements as well as the receiver depth and ray path.

Because the frequency shift alters the CIR, it should affect the estimation of CIR using the aforementioned techniques, in particular, the grid-free compressive TDE using common time delay owing to its exploitation of consistency in CIR over the multiple measurements. Thus, before its application to in situ data, its performance is examined using ray-based simulated data.

**Figure 10:**
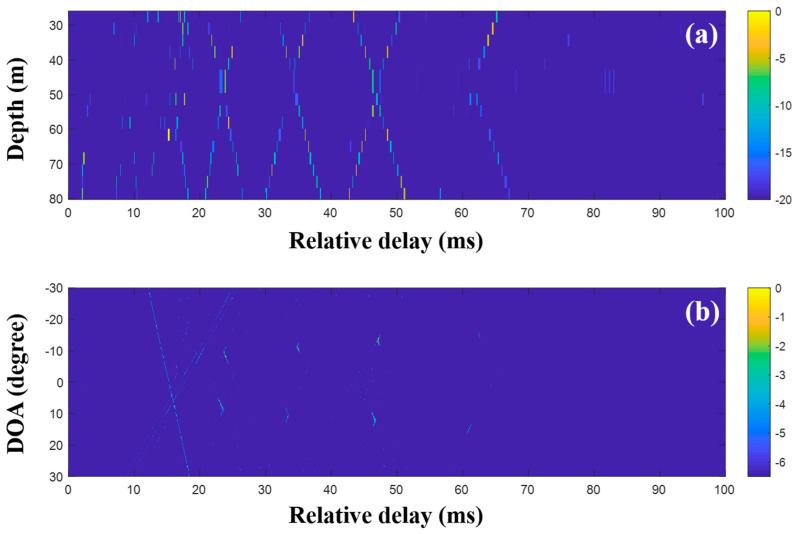
Direction-of-arrival (DOA) estimation using expanded compressive sensing (CS) with multiple measurements when the Doppler effect occurs. The signal-to-noise ratio (SNR) is −9.8 dB: (**a**) arrival structures via sequential CS usage with the signals at the vertical line array (VLA). The compressive time delay estimation (TDE) using the common time delays of the arrivals over the multiple measurements is applied. (**b**) DOAs using delay-and-sum beamforming with the arrival structures.

Figure 10 shows the arrival structures and the corresponding DOAs using the grid-free compressive TDE with common arrival times when the Doppler effect occurs during the measurement. Here, a part of the source spectrum (20–22 kHz) is used to evaluate the CIR owing to the computational burden on memory. While the high-frequency source is transmitted, the same array aperture for the low-frequency case is used for the simulation. The SNR is −9.8 dB. The arrival time delay and amplitude for a specific path are estimated differently according to each measurement, owing to the random frequency shift. However, the compressive TDE can detect the representative time delay for the specific arrival path over the multiple measurements, which results in clear arrival structures. Note that the higher resolution of the CIR is achieved with more delta-like autocorrelation of the high-frequency broadband LFM signal, which enhances DOA detection with the CIR from the compressive TDE.

## 4. SAVEX15: Experimental Results

The time domain beamforming with CS was applied to the SAVEX15 experimental data, which were gathered in the northeastern East China Sea [23,24]. The experiment site is shallow with an almost-flat bottom and a depth of 100 m. Surprisingly, a sound channel whose minimum sound speed is located near the middle of the water column and which usually exists in deep water, was observed during the experiment. The following two different transmitted-signal frequency bands were used: low (0.5–2 kHz) and high (11–31 kHz) frequencies. The transmitted signals from the source were measured using the moored VLA that is composed of 16 hydrophones with an aperture of 3.75 m, covering the water depth from 25–81 m.

The delay-and-sum beamforming with the single- and multiple-snapshot CS separately estimated the DOAs for both the low- and high-frequency cases, and they were compared with the MF beamforming results to demonstrate the CS denoising performance.

### 4.1. Low Frequency: 0.5–2 kHz

For the low-frequency case, the source was towed at the depth of 50 m, and its distance from the VLA varies from 1.5 to 3.5 km [23]. The communication signals were sequentially emitted from the source after the transmission of an LFM chirp probe signal from 0.5 to 2 kHz. Here, the probe signal was used to estimate the DOAs. Note that the probe signal was transmitted repeatedly with a pause, owing to the subsequent communication transmissions: Consecutive measures of the CIRs at the VLA were unavailable for the low-frequency case, thus a single snapshot was used for the DOA estimation. At the low frequency, the similarity of source spectrum with mean noise spectrum over the sensors is 0.46, which is calculated using inner product after their normalization. Here, the noise used for the similarity calculation is the signal part long after the main arrivals (source signals) have passed. 

**Figure 11:**
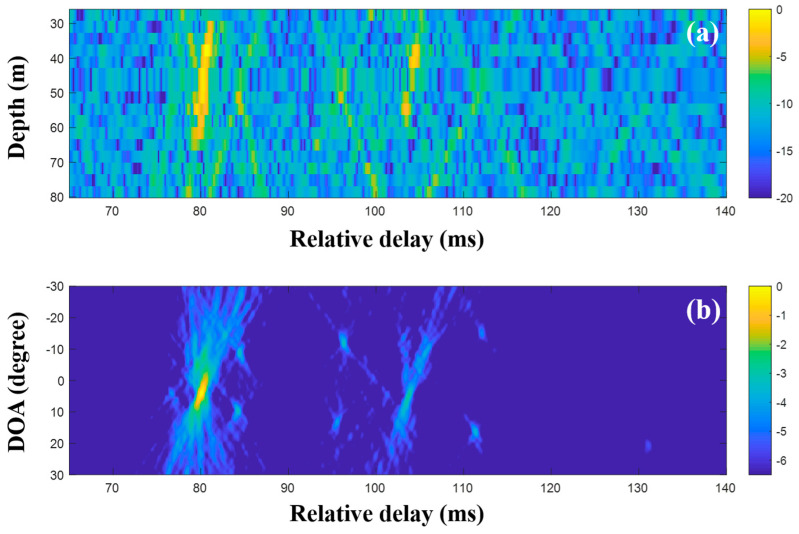
Direction-of-arrival (DOA) estimation using the matched filter (MF) with a single snapshot captured by the vertical line array (VLA) in SAVEX15 (0.5–2 kHz): (**a**) arrival structures via the application of the MF to the signals at the VLA and (**b**) DOAs using delay-and-sum beamforming with the arrival structures.

First, the MF was applied to the raw data (200-ms time signals) captured by the sensors in the VLA. Figure 11 shows a part of the CIRs from the MF with the single measurement and the corresponding DOAs. In the earlier part of Figure 11a, the strong arrivals trapped by the sound channel are observed as in the simulation. While the noise in the data should be suppressed by the cross-correlation in the MF, it blurred the arrivals, in particular, the later weak arrivals, owing to the correlated noise with transmitted signal. Thus, the arrival structures forming the x-shape were weakened over time. The beamforming reinforced the DOAs related to the arrival structures, as shown in Figure 11b.

**Figure 12:**
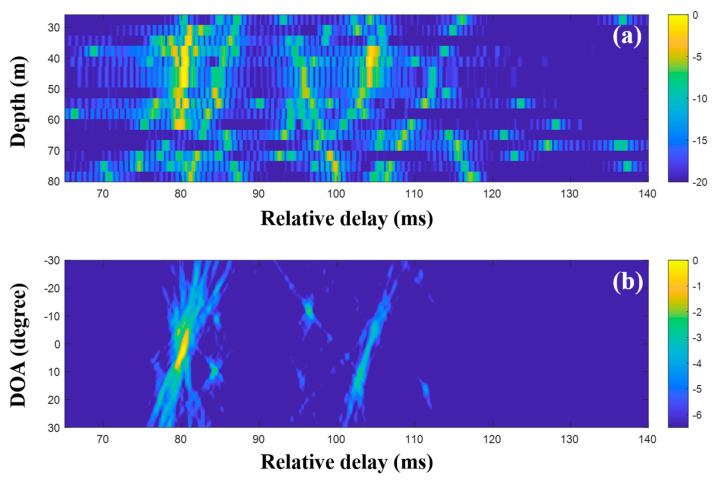
Direction-of-arrival (DOA) estimation using compressive sensing (CS) with a single snapshot captured by the vertical line array (VLA) in SAVEX15 (0.5–2 kHz): (**a**) arrival structures via sequential CS usage to the signals at the VLA, and (**b**) DOAs using delay-and-sum beamforming with the arrival structures.

In the low-frequency case, the DOAs were revealed with the CIRs from the MF, and the proposed technique using CS is not necessary. However, to examine the feasibility of the DOA detection with CS, time domain beamforming with the compressive TDE based on Equations (9) and (10) was applied to the same data, as shown in Figure 12. Here, the desired number of arrivals was set to 15. In the comparison of Figure 12 with Figure 11, while parts of the arrivals forming the x-shape are absent owing to the imperfect recovery of the CIRs by the major arrival selection, Figure 12a shows the greater noise removal of CS sparse estimation. The DOAs were revealed after the beamforming with the CIRs at the VLA. The DOAs corresponding to the weak arrivals (e.g., arrivals in the later part) were diminished or had disappeared during the denoising. The problem can be alleviated by the exploitation of multiple measurements, which is not available at the low frequency, as mentioned previously.

### 4.2. High Frequency: 11–31 kHz

To measure the variability of the CIRs owing to dynamic ocean environmental factors such as traveling surface wave, an LFM signal of 11–31 kHz with a duration of 60 ms was emitted from an 8-element vertical source array spanning a depth from 20 to 72.5 m. The source signal was sequentially transmitted from the transducers in the source array, and the CIRs were measured using the same receiver array used for the low-frequency case. Unlike the low frequency case, the source is fixed, preventing a significant frequency shift by the Doppler effect. The source and receiver arrays are approximately 2.78 km apart. Recall that the aperture of the receiver array is 3.75 m, which is much larger than half the source frequency wavelength, restricting (narrowband) frequency domain beamforming for DOA detection. Alternatively, the time domain beamforming in this work can be applied through the exploitation of the properties of the broadband source signal.

The acoustic signals from the source array were continuously recorded for one minute at eight different source depths, which enabled DOA estimation with multiple measurements for both the MF and CS. The CIRs were evaluated at intervals of the source signal duration (i.e., 60 ms) for each sensor at the receiver array. Here, ten consecutive source signals emitted from the transducer at 57.5 m were used to examine the performance of the time domain DOA estimation with CS, which can be considered to be steady owing to the short-term measurement (less than one second) and the calm sea state. At the high frequency, the similarity between source and noise spectra is 0.04, much less than that at the low frequency.

#### 4.2.1. DOA Detection Using MF with Single and Multiple Measurements

**Figure 13:**
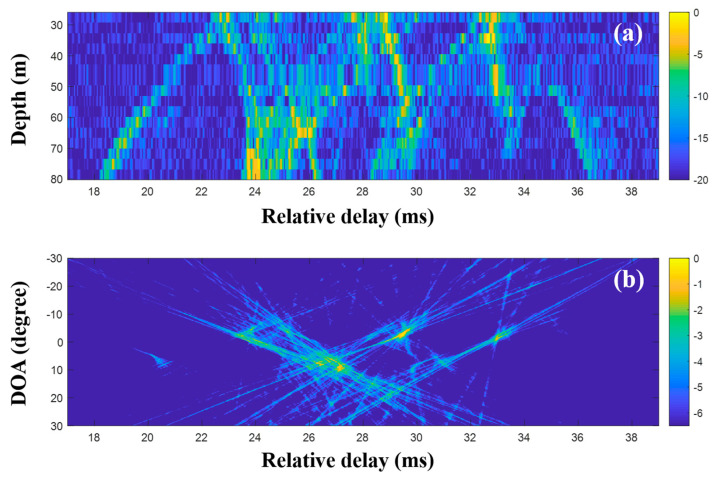
Direction-of-arrival (DOA) estimation using the matched filter (MF) with a single snapshot captured by the vertical line array (VLA) in SAVEX15 (11–31 kHz): (**a**) arrival structures via the application of the MF to the signals at the VLA and (**b**) DOAs using delay-and-sum beamforming with the arrival structures.

The CIRs according to the receiver depth and the related DOAs were estimated using the MF with single and multiple measurements, respectively. Figure 13 shows the CIRs and DOAs for which the MF was used with a single measurement. The noise after the arrivals is more suppressed than that of the low frequency case due to its lower correlation with the transmitted signal. The x-shape arrival structures in Figure 11 disappeared, owing to the water current in the experimental site that tilted the receiver array and distorted the arrival structures [30,31] (Refer to simulated CIRs in Figure 4 of reference [30], which confirm the acoustic structure change by the current). Here, the focus is on clear DOA detection in the time domain—the effects of the current on DOAs are not of interest—while the DOAs should be estimated with bias. After the application of the delay-and-sum beamforming to the CIRs, DOAs were revealed with high ambiguities (in particular, the DOAs between 22 and 30 ms), owing to side lobes in the compressed pulse near the arrivals; these made adjacent arrivals indistinguishable (in particular, at deep water depth around 24 ms in Figure 13a), smeared the arrival structures, and prevented unambiguous DOA detection.

**Figure 14:**
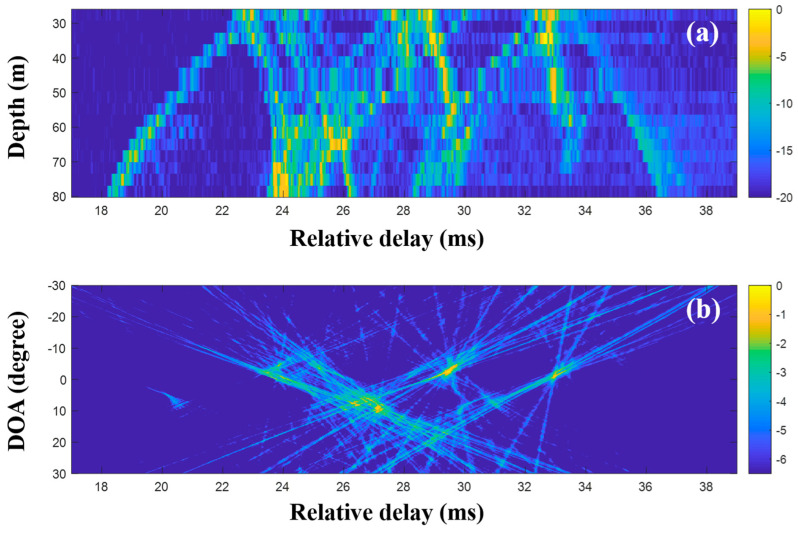
Direction-of-arrival (DOA) estimation using the matched filter (MF) with multiple snapshots captured by the vertical line array (VLA) in SAVEX15 (11–31 kHz): (**a**) arrival structures via the application of the MF to the signals at the VLA, and (**b**) DOAs using delay-and-sum beamforming with the arrival structures.

The CIRs were estimated using the MF of consecutively measured single snapshots, and they were averaged in the DOA estimation. Figure 14 shows the averaged CIRs and the DOA estimation with the delay-and-sum beamforming for which the CIRs were used; while the arrivals are more evident owing to the noise reduction, the adjacent arrivals are still clustered, leading to the indistinct arrival structures. The multiple measurement data are not significantly helpful for clear DOA detection in MF beamforming.

#### 4.2.2. Detection of DOAs Using CS with Single and Multiple Measurements

**Figure 15:**
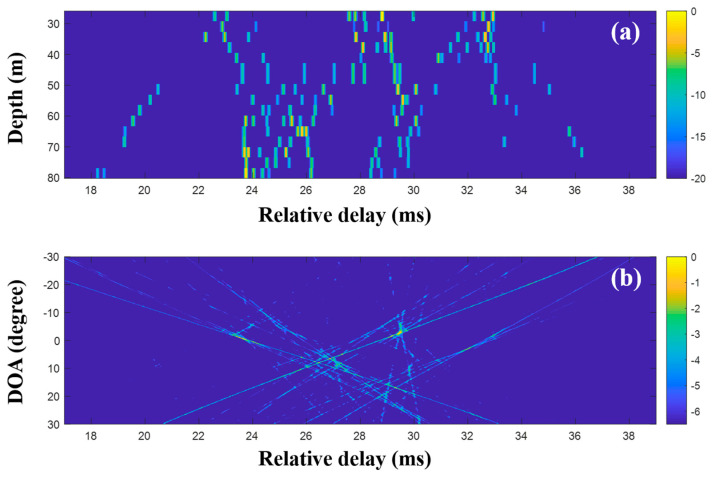
Direction-of-arrival (DOA) estimation using compressive sensing (CS) with a single snapshot captured by the vertical line array (VLA) in SAVEX15 (11–31 kHz): (**a**) arrival structures via sequential CS usage to the signals at the VLA, and (**b**) DOAs using delay-and-sum beamforming with the arrival structures.

**Figure 16:**
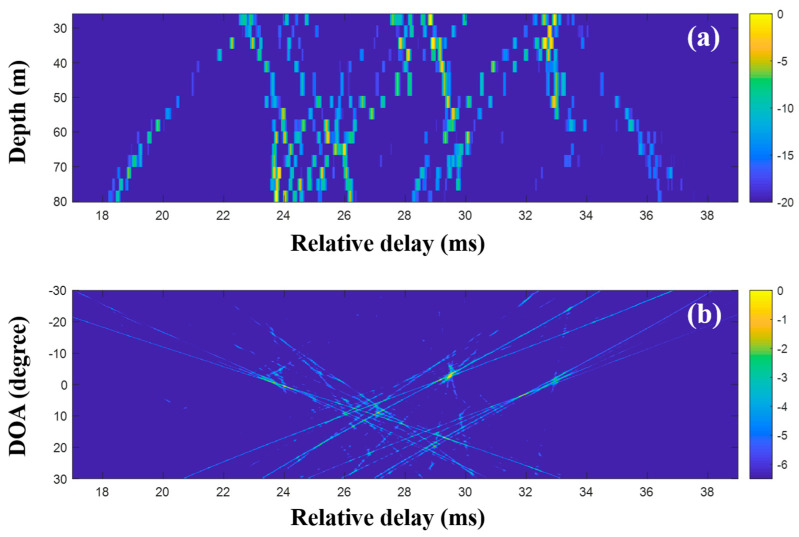
Direction-of-arrival (DOA) estimation using compressive sensing (CS) with multiple snapshots captured by the vertical line array (VLA) in SAVEX15 (11–31 kHz): (**a**) arrival structures via sequential CS usage applied to the signals at the VLA and (**b**) DOAs using delay-and-sum beamforming with the arrival structures.

**Figure 17:**
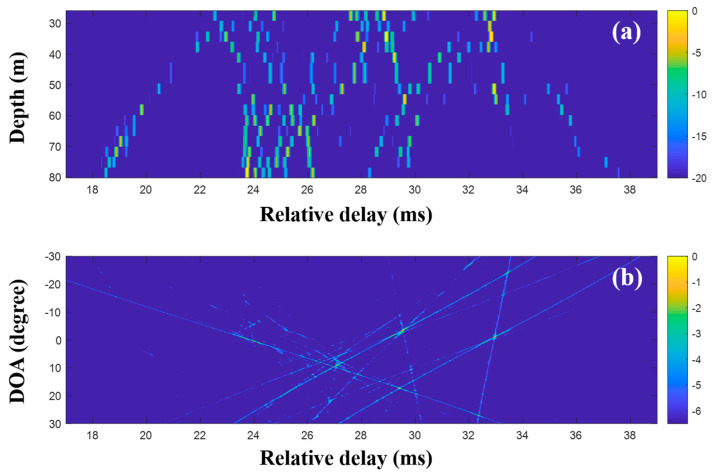
Direction-of-arrival (DOA) estimation using extended compressive sensing (CS) with multiple snapshots captured by the vertical line array (VLA) in SAVEX15 (11–31 kHz): (**a**) arrival structures via sequential CS usage applied to the signals at the VLA and (**b**) DOAs using the delay-and-sum beamforming with the arrival structures.

To suppress ambiguities, the DOAs were evaluated with the CIRs from CS using a single and multiple snapshots (Figure 15, Figure 16 and Figure 17). As in the simulation for the high frequency case, a particular part of the source spectrum was used. The desired number of arrivals was set to ten. Owing to the noise reduction by the selection in CS, the MF side lobes around the actual arrivals were notably removed, leading to a more obvious DOA estimation (Figure 15). The cluttered patterns in Figure 13b and Figure 14b were diminished, assuring that the DOAs appeared at specific spots. During the compressive TDE denoising, however, parts of the actual arrivals were missed and the associated DOAs (in particular, those around 25 ms) were weakened, owing to the insufficient coherent sum for the DOAs, such that the ambiguities remained. To mitigate these problems, multiple measurement data were exploited for the beamforming with the CS.

**Figure 18:**
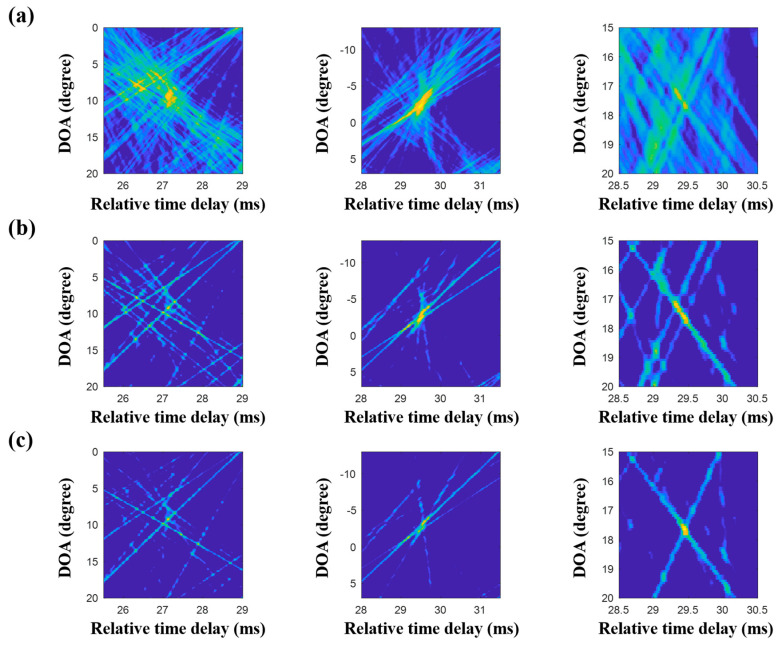
Enlarged parts of the results of delay-and-sum beamforming from three different schemes that were applied to estimate the CIRs at the array using multiple measurements: (**a**) matched filter (MF), (**b**) compressive sensing (CS) using the time delay for each single snapshot, and (**c**) CS using the time delays over multiple snapshots. The ambiguities decrease as the techniques become more sophisticated.

CS using the single snapshot was applied individually to the multiple measurements, and the CIRs from CS were averaged to reduce the noise effects on the DOA estimation. Figure 16 shows the averaged CIRs and corresponding DOAs. Note that the indistinguishable arrivals in the MF CIRs were seen separately in the CS CIRs, and the missing parts were recovered by multiple measurement estimation; the incoming acoustic wave from the upward direction around 27 ms appeared more distinctly, and the corresponding DOA was detected with less ambiguity (see the first column of Figure 18). As expected, the clearer arrival structures were beneficial for the DOA detection, including the invisible DOAs near 25 ms in Figure 15b. As in the simulation, the average via the multiple measurement technique is more effective for the CS DOA detection.

The extended compressive TDE for the multiple measurements was applied, and the common arrival time delays over the observed time period were evaluated. The contributing arrivals were derived using the common time delays according to the single snapshot composed of the multiple measurements, and they were then averaged. Figure 17a shows the production of less-scattered signals, thereby resulting in the more regular patterns of the arrival structures. Accordingly, the DOAs from the CIR-using delay-and-sum beamforming are more refined (Figure 17b). In the comparison of the final results with Figure 13 (or Figure 14), the DOAs show more certainty and are seen clearly with high resolutions; this enables the ranking of the DOAs according to priority. Parts of the DOA results (in particular, the regions around the highly ranked DOAs) from the techniques described in this work are enlarged, as shown in Figure 18. As the techniques became more sophisticated, the ambiguities of the DOA results became more suppressed, and this enabled a clearer DOA detection.

As mentioned before, the MF is generally used with proper source waveforms including an LFM signal in estimating a CIR, owing to its pulse compression and noise suppression. However, its application is limited by insufficient time resolution for separating close arrivals and non-filtered noise (Figure 13 and Figure 14). To enhance the time resolution as well as noise suppression, CS can be applied for the CIR estimation. While sparse arrivals from the CS-based CIR estimator are beneficial for obtaining a clear CIR, it misses weak arrivals owing to the sparsity when noise is present (Figure 15). To mitigate the problem, multiple measurements are exploited (Figure 16). In particular, the extended grid-free CS can find common time delays over the multiple measurements, which enable more apparent CIR estimation (Figure 17). The more refined CIRs along sensors in an array lead to more clear DOA detection (Figure 18). 

### 4.3. Discussion

As shown in the beamforming results of Section 4, the CIRs from the MF are sufficient to show the clear arrival structures, and the DOAs estimated using the CIRs combined with the delay-and-sum beamforming were clearly evident when the data were measured by the array with a sufficient SNR. However, ocean noise deteriorated the MF beamforming performance because the random noise signals emerged significantly in the CIRs from the MF, thereby increasing the ambiguities in the DOA detection.

The compressive TDE sequential usage in the continuous and discrete time domains suppressed ocean noise in the received signal owing to the selection of the actual arrivals, enabling a clearer DOA detection even when the data were contaminated by ocean noise. Furthermore, CS used the consecutive measurement more effectively owing to its ability to exploit the commonality over the multiple measurements. Figure 18 shows the more effective removal of the blurred structures in the DOA results with the usage of the more sophisticated schemes.

## 5. Conclusions

The compressive sensing (CS) technique was applied for the extraction of the channel impulse responses (CIRs) along the ocean depths. The CIRs were then used with delay-and-sum beamforming for the estimation of the direction-of-arrivals (DOAs) in the time domain. In general, the matched filter (MF) is used to evaluate the CIRs combined with the beamforming; however, when the data are contaminated by ocean noise, the MF fictitious arrivals smear the arrival structures and prevent unambiguous DOA detection. In this work, to alleviate this problem, CS is used with the following two steps (see Figure 7): (1) The grid-free compressive time delay estimation (TDE) finds time delays of the candidates for the actual arrivals; and (2) Conventional CS uses the time delay that chooses the contributing arrivals of the received signals at the array. The refined CIRs provided more obvious DOAs, while the weak arrivals and the associated DOAs were weakened or lost. A possible solution is the exploitation of multiple measurements that is beneficial for time domain beamforming with both the MF and CS. In particular, the compressive TDE was extended to search for the common time delays of the arrivals over the multiple measurements, leading to a more definitive DOA estimation.

The performance of the MF and CS beamforming were examined using simulations according to the single and multiple measurements. The beamforming schemes were then applied to the SAVEX15 data. At the low frequency, the MF beamforming is sufficient for DOA detection, whereas the side lobes around the MF arrival structures resulted in the DOA result blurring at the high frequency. CS was used with single and multiple snapshots to suppress the CIR noise. As the beamforming schemes were advanced, the DOAs were obtained with greater certainty.
